# Case Report: Convalescent Plasma, a Targeted Therapy for Patients with CVID and Severe COVID-19

**DOI:** 10.3389/fimmu.2020.596761

**Published:** 2020-11-20

**Authors:** Karel F. A. Van Damme, Simon Tavernier, Nele Van Roy, Elisabeth De Leeuw, Jozefien Declercq, Cédric Bosteels, Bastiaan Maes, Marieke De Bruyne, Delfien Bogaert, Victor Bosteels, Levi Hoste, Leslie Naesens, Piet Maes, Alba Grifoni, Daniela Weiskopf, Alessandro Sette, Pieter Depuydt, Eva Van Braeckel, Filomeen Haerynck, Bart N. Lambrecht

**Affiliations:** ^1^ Department of Internal Medicine and Pediatrics, Ghent University, Ghent, Belgium; ^2^ Laboratory of Immunoregulation and Mucosal Immunology, VIB-UGent Center for Inflammation Research, Ghent, Belgium; ^3^ Unit of Molecular Signal Transduction in Inflammation, VIB-UGent Center for Inflammation Research, Ghent, Belgium; ^4^ Center for Medical Genetics, Ghent University Hospital, Ghent, Belgium; ^5^ Primary Immunodeficiency Research Lab, Jeffrey Modell Diagnosis and Research Center, Ghent University Hospital, Ghent, Belgium; ^6^ Laboratory of ER Stress and Inflammation, VIB-UGent Center for Inflammation Research, Ghent, Belgium; ^7^ Department of Microbiology, Immunology, and Transplantation, Rega Institute for Medical Research, Division of Clinical and Epidemiological Virology, KU Leuven, Leuven, Belgium; ^8^ Center for Infectious Disease and Vaccine Research, La Jolla Institute for Immunology, La Jolla, CA, United States; ^9^ Department of Medicine, Division of Infectious Diseases and Global Public Health, University of California, San Diego (UCSD), CA, United States; ^10^ Department of Intensive Care, Ghent University Hospital, Ghent, Belgium; ^11^ Department of Respiratory Medicine, Ghent University Hospital, Ghent, Belgium; ^12^ Department of Pulmonary Medicine, Erasmus Medical Center, Rotterdam, Netherlands

**Keywords:** convalescent plasma, COVID-19, common variable immunodeficiency disorders, immunodeficiencies, case report

## Abstract

The disease course of COVID-19 in patients with immunodeficiencies is unclear, as well as the optimal therapeutic strategy. We report a case of a 37-year old male with common variable immunodeficiency disorder and a severe SARS-CoV-2 infection. After administration of convalescent plasma, the patient’s condition improved rapidly. Despite clinical recovery, viral RNA remained detectable up to 60 days after onset of symptoms. We propose that convalescent plasma might be considered as a treatment option in patients with CVID and severe COVID-19. In addition, in patients with immunodeficiencies, a different clinical course is possible, with prolonged viral shedding.

## Introduction

Coronavirus disease 2019 (COVID-19), caused by severe acute respiratory syndrome coronavirus 2 (SARS-CoV-2), was first identified in Wuhan, China in December 2019 and has since evolved into a pandemic. The majority of COVID-19 patients experience mild symptoms and recover spontaneously. In some patients however, infection may lead to severe hypoxemia, acute respiratory distress syndrome (ARDS) and death.

Next to antiviral and immunomodulatory agents, convalescent plasma might be a therapeutic option, as it has previously been used for several emerging infectious diseases, including SARS-CoV (1) and Middle Eastern Respiratory Syndrome (MERS) (2). In COVID-19, two randomized trials with convalescent plasma failed to demonstrate benefit so far, although both were stopped early due to insufficient patient enrolment and the presence of anti-SARS-CoV-2 antibodies, respectively (3, 4).

COVID-19 patients with primary and secondary immunodeficiencies might be ideal candidates for passive immunization, since a proportion of these patients will be unable to mount adequate antiviral responses to SARS-CoV-2. Only a few descriptions of patients with humoral immunodeficiencies and COVID-19 infection have been published (5–8). None of these patients received convalescent plasma.

## Case Description

We report a case of life-threatening COVID-19 in a 37-year-old man with a marked decrease in all immunoglobulin classes and a Bruton-like early B cell development block with nearly absent B cells. The patient was born in 1983 and is the child of Turkish parents. From childhood on, this patient suffered from recurring upper and lower respiratory tract infections. At adult age, the patient developed mild chronic lung disease characterized by bronchiectatic changes (for a detailed clinical and immunological examination, see **Supplementary Table 1**). The familial pedigree did not reveal a Mendelian inheritance of immunodeficiency and there was no reported consanguinity. Whole exome sequencing was performed and analysis of known primary immunodeficiency genes failed to detect disease causing variants (see **Supplementary Table 2**). Common variable immunodeficiency (CVID) was diagnosed and the patient has been receiving weekly subcutaneous injections with immunoglobulins (Hizentra) for 13 years, which led to a significant reduction of severe respiratory tract infections.

**Figure 1:**
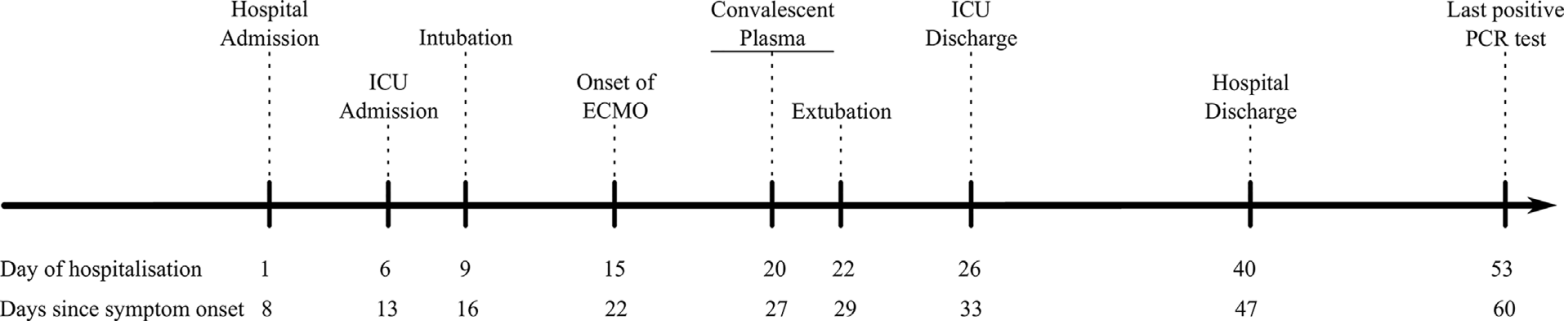
Timeline of the hospitalisation.

**Figure 2:**
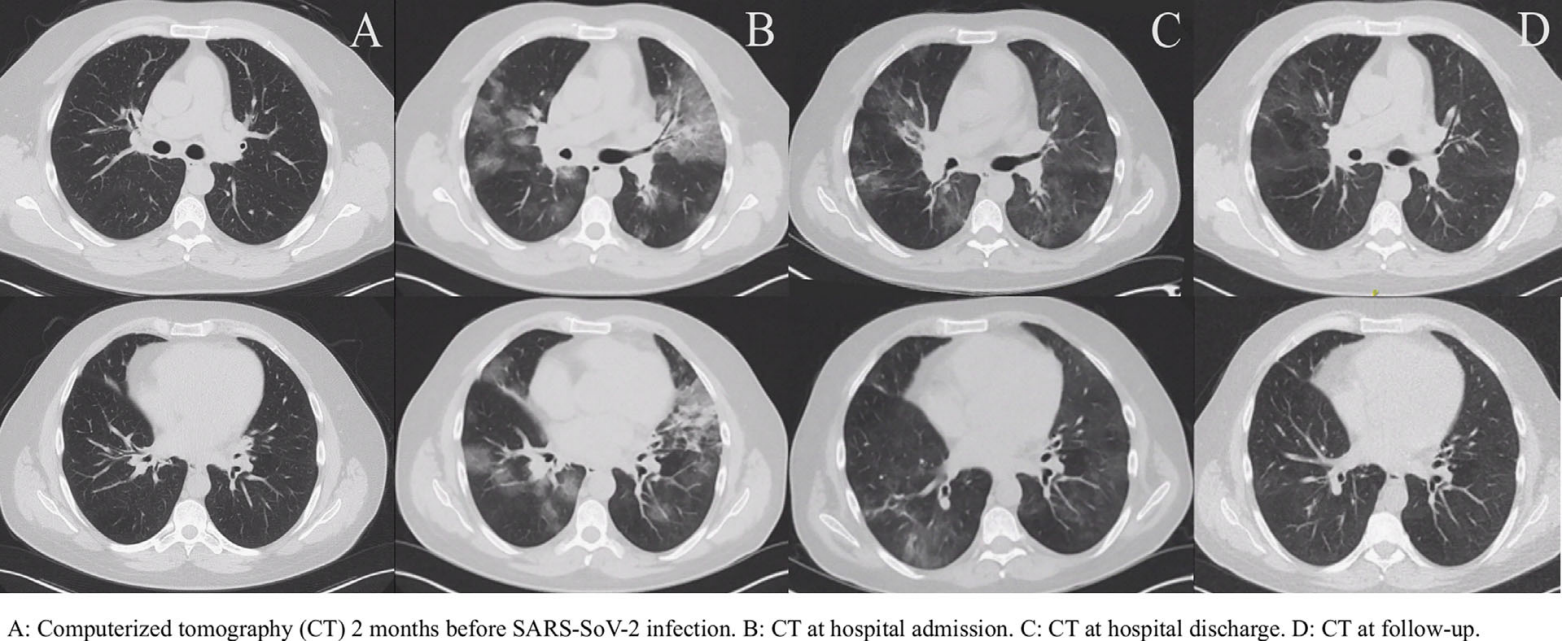
Complete resolution of the pulmonary infiltrates following recovery. **(A)** Computerized tomography (CT) 2 months before SARS-SoV-2 infection. **(B)** CT at hospital admission. **(C)** CT at hospital discharge. **(D)** CT at follow-up.

At the first peak of the COVID-19 epidemic in Belgium, the patient presented at the emergency department with fever, anorexia and a non-productive cough for 8 days (see **Figure 1**). Family members reported similar symptoms. Shortness of breath had developed 2 days prior to presentation and physical examination revealed inspiratory crepitations over both lungs. The initial work-up showed elevated CRP, ferritin, lactate dehydrogenase and D-dimer levels, without detectable eosinophils and a normal lymphocyte count (see **Supplementary Table 3**). Arterial blood gas showed mild hypoxemic respiratory failure, corresponding to an Alveolar-arterial (Aa)-gradient of 34,4 mm Hg (expected gradient of 13.3 mm Hg). An initial chest CT scan revealed bilateral ground-glass opacities (see **Figure 2**). Given the ongoing pandemic and a typical presentation, COVID-19 was suspected and his nasopharyngeal swab tested positive for SARS-CoV-2.

The patient was admitted to the COVID-19 ward and empirical treatment with antibiotics and hydroxychloroquine was initiated according to the then applicable guidelines. He was included in a clinical trial and randomized to the standard of care arm (9). After 3 days of hospitalization, the patient had unremitting fever and his respiratory status progressively deteriorated. On day 6 of hospitalization, the patient was transferred to the intensive care unit (ICU) with a severely elevated Aa-gradient of 180.3 mm Hg. The patient was intubated on day 9 of hospitalization. As permitted by the clinical trial protocol, intravenous sargramostim was initiated in addition to broad-spectrum antibiotics, methylprednisolone, prone ventilation and inhaled nitric oxide, which could only temporarily improve oxygenation. Ultimately, extracorporeal membrane oxygenation (ECMO) was started after 15 days of hospitalization.

Given the consistent deterioration, persistent detection of viral RNA, unremitting fever and underlying B cell defect, treatment with convalescent plasma was considered. After approval of the local ethical committee, on day 20 of hospitalization, the patient was transfused with 460 ml convalescent plasma. The donor was a healthy 31 year old male who had complete resolution of symptoms after a confirmed, mild SARS-CoV-2 infection. The transfusion was well tolerated and no adverse reaction was observed.

Following this treatment, the patient became independent of ECMO within one day and was successfully weaned from mechanical ventilation within two days. The SARS-CoV-2–specific neutralizing antibody titers slightly increased following the transfusion, but remained low (see **Supplementary Table 3**). The patient was transferred from the ICU to the COVID-19 ward on day 26 of hospitalisation, and another 7 days later he was discharged in good condition. The immunoglobulin substitution was continued at 12 g per week subcutaneously and azithromycin thrice weekly was initiated. Viral RNA remained detectable up to day 60 since symptom onset.

**Figure 3:**
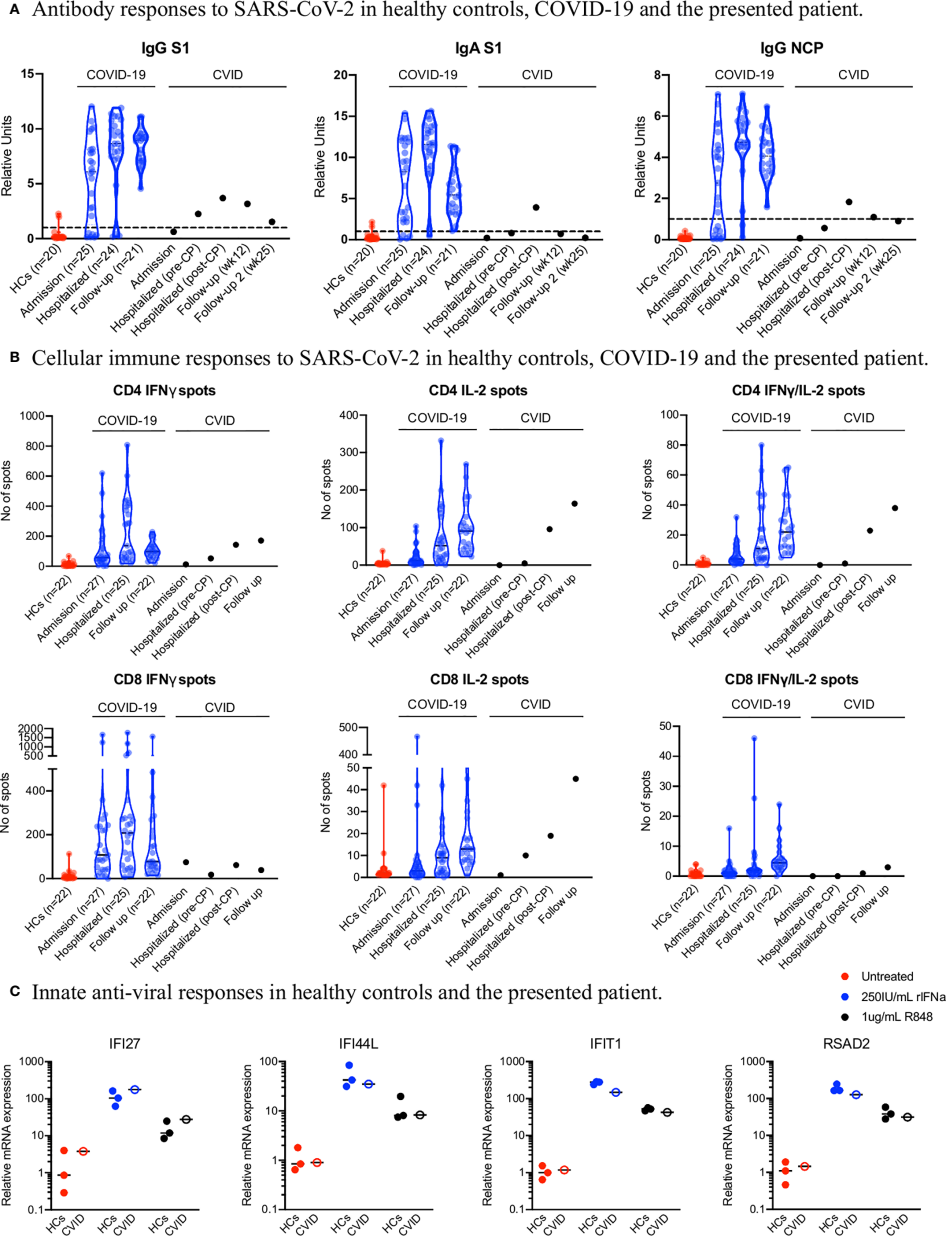
Immune Responses to SARS-CoV-2.

Immunological work-up revealed an impaired, but not completely abolished humoral response to SARS-CoV-2. Throughout the disease course SARS-CoV-2 spike S1 protein-specific IgA and nucleocapsidprotein-specific IgG remained negative, with a modest increase following the transfusion of convalescent plasma, which was comparable with the increase in antibodies reported elsewhere (10). In contrast, a reduced and delayed SARS-CoV-2 spike S1 protein-specific IgG could be detected, decreasing again at 25 weeks (see **Figure 3A**). In parallel, we studied cellular immune responses. SARS-CoV-2–specific cytotoxic lymphocytes were low throughout the time course, which was comparable with the control population. The patient generated a strong IL-2/IFNγ double-positive polyfunctional CD4^+^ T cell memory response and a IL-2/IFNγ CD8^+^ T cell response comparable to a reference population (see **Figure 3B**). Finally, we also tested the innate antiviral response and found a comparable interferon stimulated gene induction upon TLR7 and recombinant interferon-α (**Figure 3C**). These results indicated that the immunodeficiency was restricted to an impaired humoral response towards SARS-CoV-2.

## Discussion

Two main conclusions can be drawn from this case. First, the use of convalescent plasma might be considered as a therapeutic option in immunodeficient patients with severe COVID-19. Following the transfusion of convalescent plasma in this patient with a humoral immunodeficiency, ongoing fever and persisting detection of viral RNA, the patient could be weaned from ECMO and mechanical ventilation within 2 days. Second, a different course in SARS-CoV-2 infection can occur in patients with immunodeficiencies, with possibly prolonged viral shedding as SARS-CoV-2 RNA could be detected up to 60 days since symptom onset.

In general, the effect of convalescent plasma can be explained by two proposed mechanisms of action. First, pathogen-specific neutralizing antibodies can limit viral amplification by binding the immunogenic S protein and inhibiting cellular entry (11). In patients with immunodeficiencies, passive immunization has proven to be successful in the prevention and treatment of other viral infections, and this benefit might also be applicable for COVID-19, as illustrated here. Second, convalescent plasma has broad immunomodulatory effects, which involve the complement cascade, cytokine regulation, and other factors (11). Patients with CVID are well known to have dysregulated immune reactions and therefore immunomodulation could be beneficial in severe COVID-19.

An important concern for passive immunization is antibody-dependent enhancement (ADE). Non-neutralizing antibodies can facilitate viral cellular entry through interaction with Fc receptors on host cells, which can enhance inflammatory signalling, facilitate host cell infection, or both (11). ADE has been shown to occur *in vitro* in SARS-CoV and MERS-CoV (11, 12). A recent report also highlights pathogenic roles of antibodies in COVID-19 (13). This might explain why severe disease usually occurs around day 7 after symptom onset. Other possible concerns for wide-spread use of convalescent plasma include circulatory overload, thrombosis, infections and acute lung injury (11, 14).

Despite these theoretical concerns, as of October 2020, the current literature supports the safety of convalescent plasma (14). Its effectiveness in COVID-19 still remains to be established, as adequately powered randomized clinical trials are still lacking (15). Despite its uncontrolled nature, this case report supports the beneficial effect of convalescent plasma in patients with humoral immunodeficiencies and evidence of ongoing viral replication. To draw firm conclusions on the effect in immunodeficient patients, data from larger cohorts will be required however.

To date, little is known about the disease course of COVID-19 in patients with CVID, as conclusions are drawn from case reports (16). While patients with agammaglobulinemia experienced mild COVID-19–related symptoms (5, 6), the prolonged ventilation and extracorporeal support of this and another CVID patient underscores the heterogeneity of COVID-19 in patients with humoral immunodeficiencies (7). This clinical diversity is likely driven by specific underlying genetic defects, as for example Bruton’s tyrosine kinase (defective in some forms of agammaglobulinemia) drives FcγR-mediated cytokine production in monocytes (17, 18). Patients treated with anti-CD20 antibodies, such as rituximab and ocrelizumab, had equally diverse outcomes and it is still unclear whether anti-CD20 treatment impacts disease susceptibility or severity (16). Protracted infectivity should be considered in immunodeficient patients with SARS-CoV-2 infection. Although no virus could be cultured from a nasopharyngeal swab taken on day 74, the duration of positive results on polymerase chain reaction suggest an infectivity longer than described anywhere else.

More data on COVID-19 in immunodeficient patients are needed. For now, convalescent plasma can be considered a safe and potentially effective treatment in patients with humoral immunodeficiencies.

## Data Availability Statement

The original contributions presented in the study are included in the article/**Supplementary Material**. Further inquiries can be directed to the corresponding author.

## Ethics Statement

The studies involving human participants were reviewed and approved by Ethical Committee of the Ghent University Hospital. The patients/participants provided their written informed consent to participate in this study. Written informed consent was obtained from the individual(s) for the publication of any potentially identifiable images or data included in this article.

## Author Contributions

KVD, NVR, ST, BNL drafted the manuscript. EDL, JD, CB, BM, DB, MDB, EVB reviewed and edited the manuscript. ST, VB, LH, LN, FH performed cytokine measurements. ST, KVD, LN, LH, VB performed PBMC phenotyping. MDB, DB, ST, FH performed genetic analysis. PM performed plaque reduction neutralization tests and viral culture. AG, DW, AS provided peptide pools for T cell stimulation. PD, EVB, BNL coordinated transfusion with convalescent plasma.

## Funding

BNL is supported by a European Research Council advanced grant (ERC-2017-ADG), Grand Challenges Programs of VIB (M901BALA-GCP-COVID-19-SARPAC TRIAL, M902BALA-GCP-COVID-19-IL6-IL1 TRIAL), a concerted research initiative grant from Ghent University (BOF/GOA/028) and an Excellence of Science (EOS) research grant (G0G2318N). JD, CB, BM, VB, and ST are supported by grants from FWO. ST is supported by a university research grant (BOF-UGent). FH is supported by the Jeffrey Modell Foundation, University Hospital Ghent Spearhead Initiative for Immunology Research and a Grand Challenges Program of VIB.

## Conflict of Interest

AS is a consultant for Gritstone, Flow Pharma, Avalia.

The authors declare that the research was conducted in the absence of any commercial or financial relationships that could be construed as a potential conflict of interest.
