# Supplementary material for: Case Report: Convalescent Plasma, a Targeted Therapy for Patients with CVID and Severe COVID-19
Source: Front Immunol. 2020 Nov 20;11:596761. doi: 10.3389/fimmu.2020.596761 (PMC7714937; doi:10.3389/fimmu.2020.596761)
Supplement: Supplementary file 1 [file Table_1.pdf]

Supplementary Table 1: Clinical and immunological characterization

**Clinical manifestations**	
Respiratory tract infections	Recurrent pansinusitis
	Recurrent pneumonia
	Bilateral bronchiectatic disease
Other infections	Cholecystitis
Autoimmune disease	No clinical evidence
Granulomatous disease	No clinical evidence
Lymphoproliferation	No clinical evidence
Enteropathy	No clinical evidence
Age of onset	Child

**Immunological features**	**Value**	**Reference values**	**Year**
Immunoglobulins			
IgG	3 g/L	7-16 g/L	2020
IgG3	0,131 g/L	0,2-1,1 g/L	2007
IgA	0,4 g/L	0,83-4,07 g/L	2007
IgM	0,2 g/L	0,34-2,14 g/L	2007
Autoantibodies			
Lupus antigoagulant	Present		2020
Anticardiolipine IgG	Absent		2020
B2 GP1 IgG	<11 U/mL	<66 U/mL	2020
Vaccine response			
T-dependent	N/A	Under continuous SCIG	
T-independent	N/A	Under continuous SCIG	
Peripheral blood mononuclear cell immunophenotyping			
B cells	2%	6-19%	2019
Memory B cells			
IgD+ CD27+	4.0%	6,7%-25,0%	2014
IgD- CD27+	1.2%	12,5%-14,0%	2014
CD21 low B cells	3.67%	1.63%-2,56%	2014
T cells			
CD4/CD8 ratio	0.62	1-4,4	2014
CD4			
Naïve	7.7%	23,9%-46,8%	2014
CM	28.6%	31,8%-50,1%	2014
EM	41.2%	6,6%-18,4%	2014
EMRA	14.8%	0,6%-3,3%	2014
CD8			
Naïve	6.4%	13,8%-33,3%	2014
CM	2.3%	3,9%-11,1%	2014
EM	38.9%	13,0%-27,1%	2014
EMRA	52.4%	33,1%-55-1%	2014
Intracellular cytokine staining after PMA/ionomycin stimulation			
CD4			
IFNγ	62.70%	16,93%-22,38%	2020
TNF	77.90%	37,53%-47,63%	2020
IL-2	22.60%	20,55%-30,05%	2020
IL-13	0.22%	0,09%-0,19%	2020
IL-17A	0.97%	1,41%-2,60%	2020
IL-21	10.40%	1,84%-6,52%	2020
CD8			
IFNγ	89.90%	45,25%-72,83%	2020
TNF	57.50%	29,18%-45,68%	2020
NK cells			
CD56hi	0.19%	0,49%-0,79%	2014
CD16+ CD56+	19.90%	10,18%-15,40%	2014
CD16+ CD56-	4.43%	0,13%-0,24%	2014
Monocytes	11.93%	25,63%-29,83%	2014
cDCs	0.38%	0,84%-1,19%	2014
pDCs	0.21%	0,40%-0,66%	2014
iNKTs/MAITs	0.21%	2,45%-3,30%	2014
