# Supplementary material for: Case Report: Convalescent Plasma, a Targeted Therapy for Patients with CVID and Severe COVID-19
Source: Front Immunol. 2020 Nov 20;11:596761. doi: 10.3389/fimmu.2020.596761 (PMC7714937; doi:10.3389/fimmu.2020.596761)
Supplement: Supplementary file 2 [file Table_2.pdf]

Supplementary Table 2: A primary immunodeficiencies gene panel covering 499 genes revealed no likely pathogenic variants.

A: Identified variants with whole-exome sequencing.

Chr	Start	Stop	Transcript	Gene	HGVS cDNA-level	HGVS protein-level	dbSNP	gnomAD AF	gnomAD MAF	Zygosity	Classification
chr1	154436093	154436094	NM_000565.4	IL6R	c.932T>C	p.(Met311Thr)	rs757508163	0.00002093	0.0009579	heterozygous	CLASS 3
chr1	198752693	198752694	NM_002838.5	PTPRC	c.3430G>A	p.(Val1144Ile)	rs139041596	0.000174	0.001684	heterozygous	CLASS 3
chr1	235759071	235759072	NM_000081.3	LYST	c.6782G>A	p.(Arg2261His)	rs147791378	0.001365	0.0095	heterozygous	CLASS 2
chr4	56478617	56478618	NM_006947.4	SRP72	c.793G>A	p.(Val265Ile)	rs756512943	0.00000796	0.0000327	heterozygous	CLASS 2
chr5	66053675	66053676	NM_001253697.1	ERBIN	c.2357C>T	p.(Thr786Ile)	rs752498860	N/A	N/A	heterozygous	CLASS 3
chr6	31934271	31934272	NM_000063.6	C2	c.821A>G	p.(Lys274Arg)		N/A	N/A	heterozygous	CLASS 3
chr7	117531068	117531069	NM_000492.3	CFTR	c.443T>C	p.(Ile148Thr)	rs35516286	0.001671	0.00694	heterozygous	CLASS 3
chr8	99135045	99135046	NM_017890.4	VPS13B	c.1333T>G	p.(Cys445Gly)		N/A	N/A	heterozygous	CLASS 3
chr9	428369	428370	NM_203447.3	DOCK8	c.4346C>T	p.(Ser1449Leu)	rs370123223	0.00005585	0.001395	heterozygous	CLASS 3
chr11	60462415	60462416	NM_021950.3	MS4A1	c.41C>T	p.(Ala14Val)	rs568202047	0.00002481	0.0001385	heterozygous	CLASS 3
chr11	126292695	126292696	NM_148910.2	TIRAP	c.286G>A	p.(Asp96Asn)	rs8177400	0.002615	0.012	heterozygous	CLASS 2
chr12	43777662	43777663	NM_016123.3	IRAK4	c.749G>A	p.(Gly250Asp)	rs769470855	0.0000426	0.0002786	heterozygous	CLASS 3
chr13	42606498	42606499	NM_033012.3	TNFSF11	c.315T>C	p.(Gly105=)	rs146484645	0.0002302	0.001504	heterozygous	CLASS 2
chr16	10902670	10902671	NM_000246.3	CIITA	c.641G>C	p.(Ser214Thr)		N/A	N/A	heterozygous	CLASS 3
chr16	27449045	27449046	NM_181078.3	IL21R	c.1379C>G	p.(Ala460Gly)		N/A	N/A	heterozygous	CLASS 3
chr16	50711451	50711452	NM_022162.2	NOD2	c.1540T>C	p.(Tyr514His)	rs540122692	0.00004393	0.000294	heterozygous	CLASS 3
chr19	1621908	1621909	NM_003200.5	TCF3	c.882_884del	p.(Ser295del)	rs550914200	0.001983	0.003502	heterozygous	CLASS 3
chr19	50402116	50402117	NM_002691.3	POLD1	c.581C>G	p.(Ser194Cys)	rs144656348	0.0006589	0.004388	heterozygous	CLASS 2

The nomenclature of the identified variants is according to the HGVS guidelines (http://www.hgvs.org) with nucleotide 'A' of the ATG startcodon = 'c.1'.
The position of the reported variants is based on NCBI build GRCh38.

B: Gene list of 499 sequenced genes in the primary immunodeficiencies gene panel.

ACD	C1QA	CD40LG	CORO1A	ELF4	HAX1	IL2RG	LIPA	NCF4	PLEKHM1	RAG1	SERPING1	TAP1	TP53
ACP5	C1QB	CD46	CPT2	EPG5	HELLS	IL36RN	LPIN2	NCSTN	PLG	RAG2	SH2B3	TAP2	TPP1
ACTB	C1QC	CD55	CR2	ERBIN	HMOX1	IL6R	LRBA	NEIL2	PMM2	RANBP2	SH2D1A	TAPBP	TPP2
ADA	C1R	CD59	CREBBP	ERCC2	HYOU1	IL6ST	LRRC8A	NFAT5	PMS2	RASGRP1	SH3BP2	TAZ	TRAC
ADA2	C1S	CD70	CSF2RA	ERCC3	ICOS	IL7R	LTBP3	NFE2L2	PNP	RBCK1	SH3KBP1	TBK1	TRAF3
ADAM17	C2	CD79A	CSF2RB	ERCC6L2	ICOSLG	INO80	LYST	NFKB1	POLA1	RC3H1	SKIV2L	TBX1	TRAF3IP2
ADAR	C3	CD79B	CSF3R	EXTL3	IFIH1	INPP5D	MAGT1	NFKB2	POLD1	RECQL4	SLC11A1	TCF3	TREX1
AGA	C4A	CD80	CTC1	EZR	IFNAR1	INSR	MAL2	NFKBIA	POLD2	REL	SLC29A3	TCF7L1	TRIM21
AICDA	C4B	CD81	CTLA4	F12	IFNAR2	IRAK1	MALT1	NHEJ1	POLE	RELA	SLC35A1	TCIRG1	TRIM22
AIRE	C4BPA	CD86	CTPS1	FAAP24	IFNGR1	IRAK2	MAN2B1	NHP2	POLE2	RELB	SLC35C1	TCN2	TRNT1
AK2	C5	CD8A	CTSC	FADD	IFNGR2	IRAK4	MANBA	NKX2-5	POLR3A	RFX5	SLC37A4	TERC	TTC37
AKT1	C6	CDCA7	CXCL12	FAS	IGHM	IRF2BP2	MAP3K14	NLRC4	POLR3C	RFXANK	SLC39A4	TERT	TTC7A
ALG13	C6orf106	CDKN2A	CXCR4	FASLG	IGKC	IRF3	MASP1	NLRP1	POLR3F	RFXAP	SLC39A7	TFRC	TYK2
ALPI	C7	CDKN2B	CYBA	FASN	IGLL1	IRF4	MASP2	NLRP12	POMP	RHOH	SLC46A1	TGFB1	UNC119
AP1S3	C8A	CEBPE	CYBB	FAT4	IKBKB	IRF7	MBL2	NLRP3	POT1	RIPK1	SLC7A7	TGFBR1	UNC13D
AP3B1	C8B	CFB	CYBC1 (C17	FCGR1A	IKBKG	IRF8	MC2R	NLRP7	PRF1	RMRP	SMARCAL1	TGFBR2	UNC93B1
AP3D1	C8G	CFD	DBR1	FCGR2A	IKZF1	IRF9	MCM4	NOD2	PRKCD	RNASEH2A	SMARCD2	THBD	UNG
AP4E1	C9	CFH	DCLRE1B	FCGR2B	IL10	ISG15	MEFV	NOP10	PRKDC	RNASEH2B	SNX10	TICAM1	USB1
APOL1	CA2	CFHR1	DCLRE1C	FCGR3A	IL10RA	ITCH	MLPH	NRAS	PRPS1	RNASEH2C	SOCS4	TINF2	USP18
ARHGEF1	CARD11	CFHR2	DDX58	FCGR3B	IL10RB	ITGB2	MOGS	NSMCE3	PSEN1	RNF168	SP110	TIRAP	VAV1
ARPC1B	CARD14	CFHR3	DEF6	FCGRT	IL12B	ITK	MPO	OAS1	PSENEN	RNF31	SPINK5	TLR3	VAV2
ATM	CARD9	CFHR4	DGCR6	FCHO1	IL12RB1	ITPKB	MRE11	OFD1	PSMA3	RORC	SPPL2A	TLR4	VIPAS39
ATP2A2	CARMIL2	CFHR5	DGKE	FCN3	IL12RB2	JAGN1	MRTFA (MK	ORAI1	PSMB4	RPSA	SRP54	TMC6	VPS13B
ATP6AP1	CASP10	CFI	DHFR	FERMT1	IL15RA	JAK1	MS4A1	OSTM1	PSMB8	RRAS2	SRP72	TMC8	VPS33B
ATP6V0A2	CASP8	CFP	DKC1	FERMT3	IL17F	JAK2	MSH6	OTULIN	PSMB9	RSPH9	STAT1	TMEM173	VPS45
B2M	CAVIN1	CFTR	DNAJC21	FOXN1	IL17RA	JAK3	MSN	PARN	PSMG2	RTEL1	STAT2	TNFAIP3	WAS
BACH2	CBL	CHD7	DNASE1	FOXP3	IL17RC	KDM6A	MST1	PAX5	PSTPIP1	RTL1	STAT3	TNFRSF11A	WDR1
BCL10	CCBE1	CIB1	DNASE1L3	FPR1	IL18	KMT2A	MTHFD1	PBX1	PSTPIP2	SAMD9	STAT4	TNFRSF13B	WIPF1
BCL11B	CD19	CIITA	DNASE2	G6PC	IL18BP	KMT2D	MVK	PCCA	PTEN	SAMD9L	STAT5B	TNFRSF13C	WRAP53
BLK	CD247	CLCN7	DNMT3B	G6PC3	IL1RL1	KRAS	MYD88	PCCB	PTPN11	SAMHD1	STAT6	TNFRSF1A	XBP1
BLM	CD27	CLEC4D	DOCK2	G6PD	IL1RN	LACC1	MYO5A	PEPD	PTPN22	SBDS	STIM1	TNFRSF4	XIAP
BLNK	CD3D	CLEC7A	DOCK8	GATA1	IL21	LAMTOR2	MYSM1	PGM3	PTPN6	SDHA	STK4	TNFRSF9	XRCC4
BLOC1S3	CD3E	CLPB	DOK3	GATA2	IL21R	LAT	NBAS	PIGA	PTPRC	SEC61A1	STN1	TNFSF11	ZAP70
BLOC1S6	CD3G	CNBP	DTNBP1	GFI1	IL23R	LCK	NBN	PIK3CD	RAB27A	SEMA3E	STX11	TNFSF12	ZBTB24
BTK	CD4	COLEC11	EFL1	GINS1	IL2RA	LIG1	NCF1	PIK3R1	RAC2	SERAC1	STXBP2	TOP2B	ZNF341
BTLA	CD40	COPA	ELANE	HAVCR2	IL2RB	LIG4	NCF2	PLCG2					
