# Supplementary material for: Case Report: Convalescent Plasma, a Targeted Therapy for Patients with CVID and Severe COVID-19
Source: Front Immunol. 2020 Nov 20;11:596761. doi: 10.3389/fimmu.2020.596761 (PMC7714937; doi:10.3389/fimmu.2020.596761)
Supplement: Supplementary file 3 [file Table_3.pdf]

Supplementary Table 3: Laboratory results.

	Prior to admission	Admission	Admission Midcare	Intubation	Following convalescent plasma	ICU discharge	Hospital discharge	Reference range
Day of hospitalisation	-40	1	4	9	20	26	47	
Aa-gradient (mmHg)	-	34,4	-	310,1	on ECMO	104,8	-	<13,3
C-reactive protein (mg/L)	-	169,7	295	293	40,9	12,2	2,4	<5,0
Ferritin (µg/L)	-	592	1076	2874	1237	1006	179	20-280
Lactate dehydrogenase (U/L)	-	314	411	478	522	276	244	
Interleukin-6 (pg/mL)	-	-	47,25	38,99	-	-	-	<7,93
Tumor necrosis factor alpha (pg/mL)	-	-	25,91	21	-	-	-	<54,70
Monocyte chemo-attractant-1 (pg/mL)	-	-	210,05	191,25	-	-	-	16-151
sRAGE (pg/mL)	-	-	3749,5	2639,25	-	-	-	287-1473
$PRNT_{50}$ value*	-	<1/20	-	1/20	1/40**	1/80	1/80	1/640
Eosinophils (/µL)	220	0	0	50	0	160	120	28-273
Total lymphocytes (/µL)	2460	1460	1220	1010	376	830	1900	1133-3105
IgG (g/L)***	10,3	-	-	6,3	-	-	-	6-17
IgM (g/L)	<0,20	-	-	<0,20	-	-	-	0,34-2,14
IgA (g/L)	<0,28	-	-	<0,28	-	-	-	0,83-4,07

* Plaque reduction neutralization test. ** Measured 3 hours after transfusion. *** Under immunoglobulin substitution.
