# Supplementary material for: Case Report: Convalescent Plasma, a Targeted Therapy for Patients with CVID and Severe COVID-19
Source: Front Immunol. 2020 Nov 20;11:596761. doi: 10.3389/fimmu.2020.596761 (PMC7714937; doi:10.3389/fimmu.2020.596761)
Supplement: Supplementary file 4 [file DataSheet_1.docx]

**Supplementary materials and methods**

1) Whole exome-sequencing

Targeted next-generation sequencing (NGS) of the BTK gene, associated with X-linked agammaglobulinemia, was performed first. Genomic DNA was isolated from whole blood cells according to standard procedures. Primers for amplification and sequencing of the coding regions, adjacent intron-exon boundaries and UTRs of *BTK* were designed using an in-house developed primer design software program ([www.pxlence.com](http://www.pxlence.com)). Targeted NGS was performed using a flexible protocol, consisting of singleplex PCR followed by NexteraXT library preparation and sequencing on a MiSeq instrument, as previously described (1). The CLC Genomics Workbench v6 (Qiagen) was employed for read mapping against the hg19 human reference genome and variant calling. For subsequent data analysis, variants were filtered based on technical quality parameters, frequency in public population databases and *in silico* predictions. More thorough variant annotation of the remaining variants was executed using Alamut Visual software (Interactive Biosoftware) and a complementary literature search.

Subsequently, whole-exome sequencing was performed. gDNA was enriched with the SureSelectXT Low Input Human All Exon V7 kit (Agilent Technologies) followed by sequencing on a NovaSeq 6000 (Illumina). Data analysis was executed using in-house developed pipelines and limited to genes present in PID gene panel (see Supplementary Table 2). Variants were classified using an in house developed tool based on the ACMG and ACGS guidelines with adaptations. The strength of some criteria was altered under certain conditions as recommended in literature (2-5).

2) Peripheral blood mononuclear cell collection and culture

Peripheral blood mononuclear cells (PBMC) were isolated using Leucosep tubes (Greiner Bio) containing Ficoll density gradient medium. Cells were stored in Fetal Calf Serum (FCS; Sigma Aldrich; F7524) containing 10% dimethyl sulfoxide (DMSO; Sigma Aldrich; D2650) at -150°C, until further use. PBMC were thawed in 37°C preheated complete medium (RPMI-1640 medium supplemented with GlutaMAX, 10% FCS, 1% penicillin-streptomycin (Pen/Strep; 10.000U/mL; Gibco; 15140122), 1mM sodium pyruvate (Gibco; 11360070), 1% non-essential amino acids (NEAA; Gibco; 11140035) and 50μM 2-mercaptoethanol (Gibco; 31350010). In the setting of functional testing, cells were left to recuperate for 30 minutes at 37°C and 5% CO2 after removal of DMSO.

3) Flow cytometry

PBMC were stained with monoclonal antibodies labeled with fluorochromes or biotin recognizing surface markers. In general, cells were first stained with FcR block (human; Miltenyi; 130-059-901) together with biotin conjugated antibodies and Fixable Viability dye eFluor 506 (eBioscience; 65-0866-14). In a second step, remaining surface markers were stained with a mixture of antibodies in FACS buffer (DPBS pH7.4, 1% Bovine Serum Albumin, 0,05% NaN3, 1 mM EDTA) and Brilliant Stain buffer (BD Biosciences). If staining of intracellular antigens was required, cells were fixed 30 minutes in 2% paraformaldehyde at room temperature and subsequently permeabilized with FoxP3 permeabilization buffer (ebioscience; 00-5523-00). Acquisition and analysis of labeled cell suspensions was performed with a FACSymphony flow cytometer (BD biosciences) and subsequent analysis of data with FlowJo10 software (BD biosciences).

4) FluoroSpot

To quantify SARS-CoV2 specific CD4 and CD8 T cells, 0,5M PBMC were resuspended in complete medium, plated in duplicate into 96 well plates, precoated with capturing antibodies directed against IFN and IL-2. PBMC were stimulated with CD4 or CD8 T cell specific peptide pools at a final concentration of 1 μg/mL. After 23 hours of stimulation, plates were collected and spots were developed following manufacturer’s protocol (Mabtech, FSP-0102-10). Spots were revealed and quantified using Mabtech IRIS Fluorospot reader (Mabtech).

5) Interferon-stimulated gene expression

After thawing of cells, 0,5 million viable PBMC were resuspended in complete medium and transferred to 1,5mL eppendorfs. Cells were stimulated with 2 μg/mL recombinant interferon alpha or 0,5 IU/mL R848 for 3 hours at 37°C and 5% CO2. Cells were subsequently washed in PBS and lysed in 350 μL RLT buffer (1048449; Qiagen) containing 1% 2-mercaptoethanol. Lysates were stored at -80°C until further processing. RNA was obtained using the RNEasy Kit (74106; QIAGEN) following manufacturer’s instructions. Concentration and purity of RNA was assessed using the NanoDrop 8000 technology (ThermoFisher Scientific, ND-8000-GL). 500ng RNA was transcribed to cDNA using the sensifast cDNA synthesis kit (Bioline; BIO – 65054) and 15ng cDNA (estimated from input RNA) was used as input for quantitative Real-Time PCR (Lightcycler 480, Roche). Gene expression was analyzed using qbase+ software version 2.6 (Biogazelle).

6) Anti-SARS-CoV-2 ELISA

Serum was diluted at 1:100. IgG and IgA responses against the SARS-CoV-2 S1 domain of the spike protein and IgG responses to the nucleocapsid protein were measured using commercially available kits (Euroimmun EI 2606-9620 G, EI 2606-9620-2 G, EI 2606-9620 A), according to the manufacturer instructions.

7) References

1. De Leeneer K, Hellemans J, Steyaert W, et al. Flexible, scalable, and efficient targeted resequencing on a benchtop sequencer for variant detection in clinical practice. Hum Mutat. 2015 Mar;36(3):379-87.
2. Richards S, Aziz N, Bale S et al. Standards and guidelines for the interpretation of sequence variants: a joint consensus recommendation of the American College of Medical Genetics and Genomics and the Association for Molecular Pathology. Genet Med 2015;17:405-424.
3. Nykamp K, Anderson M, Powers M, et al. Sherloc: a comprehensive refinement of the ACMG-AMP variant classification criteria. Genet Med 2017;19:1105-1117.
4. Abou Tayoun A, Pesaran T, DiStefano M et al. Recommendations for interpreting the loss of function PVS& ACMG/AMP Variant Criterion. Human Mutation 2018;39:1517-1524.
5. Biesecker LG, Harrison SM. The ACMG/AMP reputable source criteria for the interpretation of sequence variants. Genet Med 2018;20:1687-1688.
6. Plagnol V, Curtis J, Epstein M, Mok KY, Stebbings E, Grigoriadou S, et al. A robust model for read count data in exome sequencing experiments and implications for copy number variant calling. Bioinformatics. 2012;28:2747–2754.
